# Quantifying Caregiver Movement when Measuring Infant Movement across a Full Day: A Case Report

**DOI:** 10.3390/s19132886

**Published:** 2019-06-29

**Authors:** Judy Zhou, Sydney Y. Schaefer, Beth A. Smith

**Affiliations:** 1 Infant Neuromotor Control Laboratory, Division of Biokinesiology and Physical Therapy, University of Southern California, 1540 Alcazar St, CHP 155, Los Angeles, CA 90089, USA; 2 Motor Rehabilitation and Learning Laboratory, School of Biological and Health Systems Engineering, Arizona State University, 1151 S Forest Ave Tempe, AZ 85287, USA; 3 Infant Neuromotor Control Laboratory, Division of Biokinesiology and Physical Therapy and Department of Pediatrics, University of Southern California, 1540 Alcazar St, CHP 155, Los Angeles, CA 90089, USA

**Keywords:** pediatrics, acceleration, leg movement, wearable sensors, movement system

## Abstract

There is interest in using wearable sensors to measure infant movement patterns and physical activity, however, this approach is confounded by caregiver motion. The purpose of this study is to estimate the extent that caregiver motion confounds wearable sensor data in full-day studies of infant leg movements. We used wearable sensors to measure leg movements of a four-month-old infant across 8.5 hours, during which the infant was handled by the caregiver in a typical manner. A researcher mimicked the actions of the caregiver with a doll. We calculated 7744 left and 7107 right leg movements for the infant and 1013 left and 1115 right “leg movements” for the doll. In this case, approximately 15% of infant leg movements can be attributed to background motion of the caregiver. This case report is the first step toward removing caregiver-produced background motion from the infant wearable sensor signal. We have estimated the size of the effect and described the activities that were related to noise in the signal. Future research can characterize the noise in detail and systematically explore different methods to remove it.

## 1. Introduction

Wearable sensors have become increasingly popular as a measure of movement characteristics and physical activity. In past infant studies, we used wearable sensors to identify the number of infant leg and arm movement bouts produced across a day in the natural environment, as well as the duration, acceleration, and type of each (e.g., unilateral or bilateral) [1,2,3]. Specific characteristics of limb movements have been shown to be related to motor development in infants and can classify infants at risk of developmental delay [1,4,5,6]. However, the effect of caregiver-produced background motion on infant wearable sensor data is unknown.

In designing studies that use wearable sensor technology to quantify an infant’s limb movements throughout a full day, our goal was to develop algorithms to screen out non-infant produced movement or noise. We created threshold-based algorithms and used video coding as the gold standard to differentiate infant leg movements from such noise. We used acceleration and angular velocity thresholds for leg movement, quantifying them so that each pause or change in direction is counted as a new movement. We validated this algorithm against manual counting from video data and found a sensitivity of 92% [1]. However, the video data used for validation only covered 20 seconds of data and did not include effects of infant–caregiver interaction. In theory, because we are using both acceleration and angular velocity to identify infant-produced movements, we should be able to filter out background acceleration such as from riding in a stroller or a car. In fact, we were able to show that our algorithm did not identify the background movement of a mechanical swing as infant movement [1]. However, we did acknowledge that movements such as a caregiver lifting an infant’s legs during a diaper change would be counted as infant-produced movement.

Using the algorithm, we quantified and characterized full-day leg movements of infants with typical development between 1–12 months of age across three separate longitudinal data collections for each infant. Infants produced, on average, around 15,000 leg movements of each leg per day. Adjusting to different lengths of sensor wear per day and different amounts of nap time, infants produced a movement rate, on average, of around 1800–1900 movements per hour awake. Because the data collection was done across a full day (8–13 hours), data encompassed a wide range of movements and infant activities [1].

Opal sensors were not created for analysis of infant movement, but in a past study we showed that sensors on infant ankles did not significantly change the frequency of movement. In 2–10-month old infants with typical development and at-risk for developmental disability, Jiang et al. (2017) found negligible difference in frequency of leg movements in infants with and without the presence of sensors on the infant’s ankles. These results were obtained using video coding [7]. From this, we can be comfortable with using wearable sensors to study infant movements. However, other factors such as caregiver motion may confound the data and the size of the effect is unknown.

Worobey et al. (2009) first addressed caregiver motion confound by using a sensor on the ankle to track a 24-hour period during which a research assistant with a toy doll imitated a mother and her infant. The sensors used in this study [Micro Mini Motionlogger (Ambulatory Monitoring, Inc., Ardsley, NY, USA)] tracked acceleration only, recording movement above a certain acceleration threshold. They did not quantify the physical activity or movement of the doll in any way. They only reported that the toy doll was measured as ‘awake’ for 632 minutes based on the presence of acceleration values above threshold [8]. As the doll itself cannot make movements, all of its activity would reflect noise or external handling. Caregiver motion is something that has not been directly considered in other full-day studies of infant leg movement, but we have the ability to quantify it here using our algorithms. Our goal in this case report is to provide a first step toward removing caregiver-produced background motion from the infant wearable sensor signal by estimating the size of the effect and describing the activities related to noise in the signal. Future research can characterize the noise in detail and systematically explore different methods to remove it.

## 2. Materials and Methods

In this study, we used wearable sensors to record leg movement data collected from a human infant and a doll, to provide a quantitative estimate of how much of the measured movement can be attributed to caregiver-produced background motion. A researcher interacted with a doll while mimicking the actions of the caregiver with the infant throughout the recording period. Collected sensor data (Opals, APDM, Inc., Portland, OR, USA) included both acceleration and angular velocity, which we analyzed to determine the number of leg movements made and the kinematic characteristics of each movement. As the doll’s legs were fixed in place and only moveable by direct action of the researcher, all movement recorded for the doll was a result of background motion caused by the researcher, stroller, or car. For this reason, we will refer to the movement calculated from the doll’s leg as “leg movements” in quotations.

This study was approved by the Institutional Review Board of University of Southern California. One female infant 4 months of age participated in the study, and her mother signed an informed consent/parental permission form before participating. The infant was typically-developing and from a singleton, full term birth. She did not have any known visual, orthopedic, or neurologic impairment at the time of data collection and did not have any observed delays in motor development (scored above the 5^th^ percentile on the Alberta Infant Motor Scale). The infant weighed 14 lbs 13 oz, with a length of 26 inches. The toy doll used for the study weighed 1 lb 15 oz with a length of 16.5 inches.

**Figure 1:**
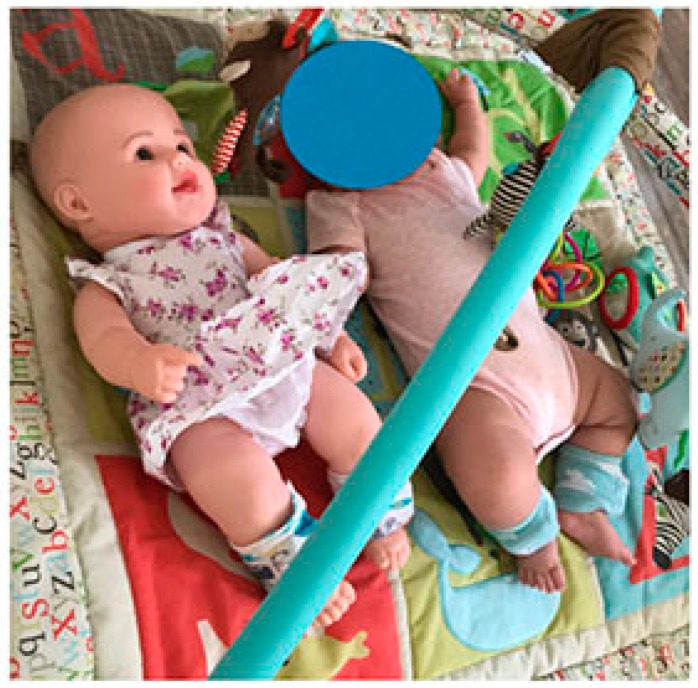
Infant and doll, both wearing sensors on each ankle.

One day of data collection of approximately 8.5 hours took place at the infant’s home and in the surrounding community. Prior to data collection, the infant’s length and weight were measured, and the Alberta Infant Motor Scale (AIMS) was administered to quantify motor development status. Movement sensors (Opals, APDM, Inc., Portland, OR, USA) were placed, one each, on the infant’s left and right ankles. As our work [1,3,4,5,6] and the work of others [8,9] has established a precedent for collecting wearable sensor data at the ankles to measure overall infant physical activity and developmental status, we placed the sensors on the ankles. Placement on the arms can be considered if arm reaching is the developmental skill of interest [2]. The sensors measured 48.5 × 36.5 × 13.5 mm and weighed 22 g. As shown in Figure 1, the sensors were attached just proximal to the ankle joints using leg warmers with pockets to secure and cover the sensor. Similar wearable sensors were placed in the same locations on the toy doll. Both sets of devices began recording movements at 9:04 and continued until 17:35. The 8.5 hours of data recorded here were consistent with our previous studies where infants wore the sensors from the morning research visit until they went to bed for the night, resulting in approximately 8–13 hours of data [1,4]. The devices collected synchronized tri-axial accelerometer and gyroscope data at 20 samples per second. Please see our previous publication [1] for full rationale for data collection and analysis procedures.

The caregiver was instructed to feed, care, play, and otherwise handle the infant as in a regular day, while the researcher mimicked all infant-caregiver interactions with the toy doll. The researcher kept a journal logging the time and duration of the day’s events throughout the duration of sensor recording. For example, the researcher laid the doll down to sleep, positioned the doll for feeding and burping, and placed the doll in the stroller or car as the caregiver did with the infant. The legs of the doll were fixed in place and only rotated at the hip joints when they were intentionally moved by the researcher. This only occurred when the caregiver moved the infant’s legs directly (such as in a diaper change) or changed the infant’s body position (such as positioning for burping). When the infant went out into the community, she was in a car seat that was in the car or stroller. When she was in the car, the doll was secured in a similar position next to the infant in the back seat. When the infant was in the stroller, the doll was placed in the same stroller near the infant. The doll and infant were at the same height from the ground throughout most of the day. The exception to this was while in the stroller, when the doll was positioned a few inches lower (closer to the ground) than the infant. This way, the infant and doll experienced the same motions of the car and stroller. Given this data collection procedure, “movements” of the doll could come from direct motion (moving the entire doll or the doll’s legs at the hip joint) or indirect motion (background acceleration due to the caregiver, car, or stroller moving).

We used MATLAB (The MathWorks, Inc., Natick, Massachusetts, USA) to calculate the number of leg movements for the infant and the “leg movements” for the toy doll, as well as the duration, average acceleration, peak acceleration, and type of each movement [1,3]. After data collection, the pattern of infant and toy doll activity was plotted on separate graphs in histogram format, displaying the resultant acceleration at each data point for visualization purposes. A larger amplitude on the plot represents greater total acceleration measured. Using the journal as a reference, these plots helped to determine what activity was taking place when the largest amplitude accelerations were observed in the doll and infant.

## 3. Results

We measured 284.4 minutes of activity for the toy doll and 461.4 minutes for the infant overall. The doll’s right and left legs produced 1013 and 1115 total “movements”, respectively, compared to 7744 and 7107 in the infant. The doll showed more bilateral “movements” than the infant (72% and 78% of all right and left leg movements, respectively, versus 57% and 56% in the infant). The infant is considered to be asleep if there are less than three leg movements in 5 minutes. A leg movement is specified as “unilateral” if only one leg is moving (either left or right), “bilateral” if while one leg is moving, the other also moves (asynchronous + synchronous), and “bilateral synchronous” if both legs are moving and start at the same time [3]. These results are shown in Table 1. 

The infant showed a higher average acceleration than the doll for both legs: 2.43 m/s^2^ for the left leg and 1.91 m/s^2^ for the right leg. In contrast, the doll’s average acceleration was 1.44 m/s^2^ for the left leg and 1.46 m/s^2^ for the right leg. Table 2 describes these results as well as the peak acceleration and standard deviation of the measurements.

**Figure 2:**
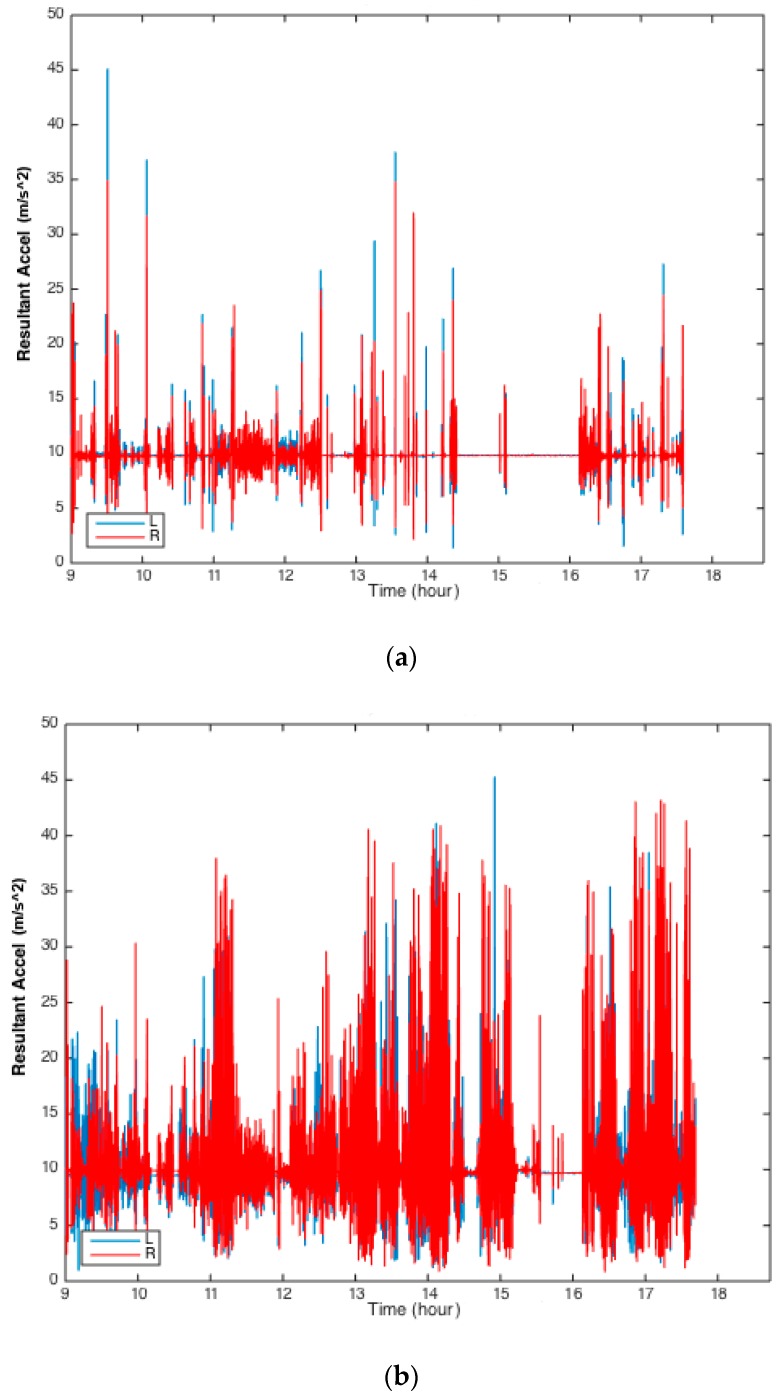
(**a**) Histogram of doll resultant raw acceleration signal from the left (L) and right (R) legs across a full day. (**b**) Histogram of infant resultant raw acceleration signal from the left (L) and right (R) legs across a full day.

**Figure 3:**
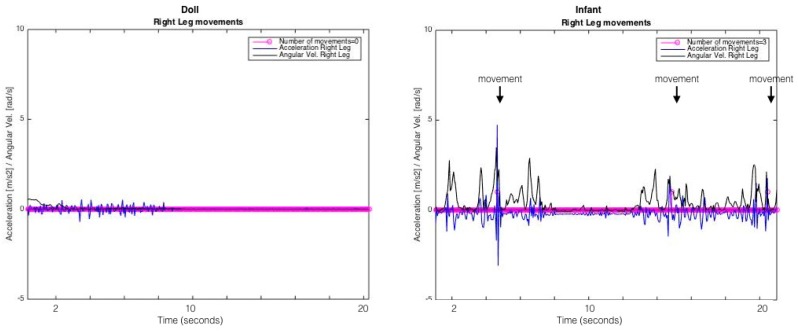
Twenty seconds of analyzed data from the right leg of the doll (**left**) and infant (**right**). The blue line shows resultant acceleration (m/s^2^) and the black line shows resultant angular velocity (rad/s). These data were recorded when the infant and doll were in the car. No movements were identified for the doll while three movements were identified for the infant (pink circles identified with additional text above the signal). Movements were identified using both acceleration and angular velocity signal thresholds as described in Smith et al., *Sensors*, 2015 [1].

The researcher kept a journal detailing the precise time of all events throughout the day, which can be seen in Table 3. Larger amplitude of acceleration can be appreciated from the doll during the following activities: sitting in a car, sitting in a stroller, and being held (Figure 2a). In contrast, larger amplitude of acceleration from the baby can be observed during these activities: laying supine in the gym, sitting, and feeding (Figure 2b). It should be noted that Figure 2a,b show only raw resultant acceleration data, however identification of leg movements per our threshold-based algorithm requires both resultant acceleration and resultant angular velocity [1]. Figure 3 shows 20 seconds of analyzed data from the right leg of the infant and the doll while in the car. Three leg movements were identified for the infant while none were identified for the doll, despite the presence of some acceleration and angular velocity signal.

## 4. Discussion

Across the 8.5 hours of sensor data, we calculated 7744 left leg and 7107 right leg movements for the infant and 1013 left leg and 1115 right leg “movements” for the doll. In this case, approximately 15% of infant leg movements can be attributed to background motion of the caregiver. These findings represent the first quantitative estimate of caregiver/background motion, although the problem was acknowledged and described in a cautionary note by Worobey and colleagues in 2009 [8].

The most notable differences between the movements of the infant and the “movements” of the doll can be observed in the type of movement and the acceleration. The toy doll had more bilateral “movements” than the infant: 78% and 72% of the time in the left leg and right leg, respectively, compared to 56% and 57% in the infant. However, this is expected because the doll’s legs, although movable at the hip joint, were fairly fixed in a constant position. The average and peak leg movement accelerations were smaller in the doll than the infant, particularly peak accelerations (see Table 2). This information may be of use in future efforts to filter out noise and caregiver motion, although it is likely that more advanced signal processing approaches (i.e., frequency analysis, machine learning) will be needed. Advanced signal processing approaches that are able to consider multiple characteristics of the signal will likely be best at differentiating caregiver and infant movement, as both types of movement likely have overlapping characteristics. For example, it is not enough to just filter out lower acceleration movements, as infants do produce small acceleration movements at times.

For the infant in this study, smaller magnitude accelerations were observed when the infant was in the car seat (see Figure 2b, between 9:20–10:45, 11:20–13:00). These data are consistent with an earlier study by Jiang et al. (2016), which found that infants in car seats showed significantly less leg movements and smaller average peak acceleration of leg movements compared with the supine position or the gym [10]. Data in Jiang et al. (2016) were collected in a stationary car seat, as opposed to our data in this study, where the car was moving at times.

There are limitations to this study. Regarding the procedure, the doll was lighter in weight and shorter in length than the infant. The doll having shorter legs that the infant would be important to consider if we were directly comparing leg movements of the infant to actual movements of the legs of the doll. The infant had a sensor at the ankle and movement at the hip and knee joints. The doll, however, was not able to bend at the knee joint and was moveable only with direct manipulation at the hip joint by the researcher. Given this data collection procedure, “leg movements” of the doll could come from direct motion (moving the entire doll or the doll’s legs at the hip joint) or indirect motion (background acceleration due to the caregiver, car, or stroller moving). The shorter length of the doll’s legs would produce smaller acceleration values than the longer legs of the infant only in cases where the doll’s legs were moved at the hip joint at the same that the infant moved her legs at her hip joint. This is not a significant concern here as the doll’s legs were manipulated by the researcher very few times across the day (see Table 3) in comparison to the thousands of leg movements measured. Most of the “leg movements” of the doll were attributed to background acceleration due to the caregiver, car, or stroller moving. The difference in weight may be of consequence. Acceleration of movements attributed to the infant were either produced by the infant or by external forces acting on the infant. In the case of the doll, acceleration of “movements” were all produced by external forces acting on the doll. As a result of the doll being lighter than the infant, the resulting accelerations produced by external actions may be different, and environmental causes of bouncing (such as hitting a bump in the road when sitting in a car seat or stroller) could more easily act on a lighter object. Additionally, the data collected from the doll were dependent on the researcher’s ability to mimic the caregiver precisely throughout the recording period. Replicating behavior precisely is a difficult task due to its biomechanical, behavioral, and observational demands. In regard to the study design, our estimate is based on a typical day for a single four-month-old infant. While it is true that the amount of caregiver-produced background noise would likely vary across different days and infant-caregiver pairs, determining that there is, for example, 12%–20% noise in the signal as opposed to the 15% we estimated here does not change the future work needed to solve the problem. We now know that around 15% of infant movement measured by wearable sensors can be attributed to background acceleration, and most of this noise can be attributed to the caregiver, car, or stroller moving. The important question now is: how do we adequately identify and remove this noise from the signal?

From a single observational data collection in the natural environment, we cannot precisely determine which caregiver motions caused the doll “movements”, nor identify and compare the effects of different activities or positions. To determine which caregiver motions produce the largest effects, and specifically measure what those effects are, a controlled study is needed where specific repeated actions (e.g., rocking, baby-carrying and baby-wearing while walking, riding in a moving stroller, etc.) are recorded simultaneously from the wearable sensors and a gold standard measurement system such as three-dimensional motion analysis or video. The most common sources of noise should be measured and accounted for in a systematic way, across multiple caregiver-infant pairs, to fully characterize and describe the noise in the signal. One potential way to do this would be to use a doll that is exactly similar to an infant in terms of size, weight, and passive movement ability and have caregivers handle it as they would their infant across standardized common noise-producing activities. In doing so, any motion recorded for the doll would be noise. Another potential way to do this would be to place sensors on the caregivers to directly measure the caregiver-produced background motion. Video-taping across the day in the natural environment could also be considered. Once the specific characteristics of the noise (non infant-produced movement) in the signal have been identified and characterized, then methods to remove it can be created.

## Figures and Tables

**Table 1:** Awake time and frequency of movements across a full day.

	Baby (Left Leg|Right Leg)		Doll (Left Leg|Right Leg)	
**Total awake time (min)**	461.4		284.4	
**Total awake time (h)**	7.7		4.7	
**Total recording time (h)**	8.5		8.5	
**Threshold acceleration (m/s^2^)**	1.15		1.1	
**Total leg movements**	7744	7107	1013	1115
**Total unilateral leg movements**	3419	3035	218	310
**Total bilateral leg movements**	4325	4072	795	805
**Total bilateral synchronous leg movements**	18		3	
**Movement rate (frequency per hour of awake time)**	1007	924	214	235
**Movement rate average (frequency per hour of awake time, both legs)**	966		224	
**Proportion unilateral movements**	0.44	0.43	0.22	0.28
**Proportion bilateral movements**	0.56	0.57	0.78	0.72

**Table 2:** Acceleration and duration of individual leg movements, average values across a full day.

	Baby (Left Leg|Right Leg)		Doll (Left Leg|Right Leg)	
**Average duration (s)**	0.26	0.27	0.30	0.28
**Standard deviation duration (s)**	0.12	0.13	0.16	0.15
**Average acceleration (m/s^2^)**	2.43	1.91	1.44	1.46
**Standard deviation of average acceleration (m/s^2^)**	1.97	1.29	0.88	0.97
**Peak acceleration (m/s^2^)**	4.94	3.79	2.74	2.77
**Standard deviation of peak acceleration (m/s^2^)**	4.88	3.10	2.18	2.33

**Table 3:** Journal of infant activity across a full day in the natural environment.

Time	Activity
9:04	sensors on
9:04–9:20	in supine
9:20–10:44	car seat (stroller–car–stroller–car (10:12–10:35 asleep))
10:44–10:55	feeding (breast)
10:55–11:00	diaper change
11:00–11:15	gym, mostly supine with some rolling into prone
11:15–11:20	held, carried, into car seat
11:20–11:50	car seat (car)
11:50–12:15	car seat (stroller)
12:15–12:30	car seat (car)
12:30–13:00	car seat (stroller)
13:00–13:15	sitting on floor
13:15–13:33	feeding (breast)
13:33–13:42	sitting in caregiver lap
13:42–14:13	gym, mostly supine with some rolling into prone
14:13–14:21	sitting on floor
14:21–14:28	held
14:28–14:44	feeding (breast)
14:44–15:06	nap attempt, held or in supine
15:06–15:09	held, soothed (crying)
15:09–16:05	nap attempt (supine), slept about 30 min
16:05–16:10	crying
16:10–16:25	held/walking/dancing
16:25–16:28	diaper change
16:28–16:45	sitting in caregiver lap
16:45–17:19	gym, mostly supine with some rolling into prone
17:19–17:35	sitting on floor
17:35	sensors off
